# Prevalence of neurodegenerative/demyelinating disorders in patients with achalasia

**DOI:** 10.1515/tnsci-2022-0249

**Published:** 2022-10-10

**Authors:** Martin Jerie, Zuzana Vackova, Zdenek Vojtech, Jan Mares, Eva Meluzinova, Jana Krajciova, Josef Vymazal, Hana Cerna, Jan Martinek

**Affiliations:** First Faculty of Medicine, Charles University, 12108 Prague, Czech Republic; Department of Neurology, Na Homolce Hospital, 15000 Prague, Czech Republic; Department of Hepatogastroenterology, Institute for Clinical and Experimental Medicine, 14021 Prague, Czech Republic; Institute of Physiology, First Faculty of Medicine, Charles University, 12108 Prague, Czech Republic; Charles University, Third Faculty of Medicine, 10000 Prague, Czech Republic; Department of Neurology, Second Faculty of Medicine, Charles University, Motol University Hospital, 15000 Prague, Czech Republic; ResTrial s.r.o., 16000 Prague, Czech Republic; Department of Radiology, Na Homolce Hospital, 15000 Prague, Czech Republic; Sarkamed s.r.o., 27401 Slany, Czech Republic

**Keywords:** achalasia, demyelinating process, neurodegeneration

## Abstract

**Introduction:**

Esophageal achalasia is a primary motility disorder. Although the exact pathogenesis is unknown, autoimmune, and neurodegenerative processes seem to be involved similarly to neurodegenerative and/or demyelinating disorders (NDDs). We hypothesized that the prevalence of NDD may be higher among patients with achalasia and vice versa as the background pathogenetic mechanisms are similar.

**Methods:**

This was a prospective, comparative questionnaire-based study. Patients with achalasia and patients with NDD were enrolled. Selected patients with achalasia were thoroughly examined by a neurologist and selected patients with NDD were examined by a gastroenterologist to confirm or rule out NDD or achalasia. We assessed the prevalence of both achalasia and NDD and compared them with their prevalence in general population.

**Results:**

A total of 150 patients with achalasia and 112 patients with NDD were enrolled. We observed an increased prevalence of NDD among patients with achalasia (6.0% (9/150); 95% CI (confidence interval): 3.1–11.2%) as compared to the estimated 2.0% prevalence in general population (*p* = 0.003). Although 32 out of 112 patients (28.6%) with NDD reported dysphagia, we did not observe significantly increased prevalence of achalasia in these patients (1.8% (2/112) vs 0.8% in general population, *p* = 0.226).

**Conclusion:**

The prevalence of NDD was significantly higher among patients with achalasia (6.0%) compared to general population (2.0%), suggesting an association of these disorders. Large-volume studies are necessary to confirm this finding.

## 1 Introduction

Achalasia is an infrequent motility disorder of the esophagus characterized by the absence of peristalsis and impaired relaxation of the lower esophageal sphincter caused by the loss of predominantly inhibitory (noncholinergic, nonadrenergic-nitrinergic) neurons [1,2]. The exact etiopathogenesis of achalasia is unknown. Current theories presume multifactorial mechanisms that include autoimmune inflammation (triggered either by genetic factors, viral infection, or both) [3,4], and neurodegeneration. A genetic component might be co-responsible for the development of achalasia as it can be part of an established genetic disorder (e.g., Allgrove syndrome, Down syndrome) [5]. Moreover, cases of familial achalasia have been reported [6].

Several neurodegenerative and/or demyelinating disorders (NDD) seem to share some similarity with pathophysiological pathways seen in achalasia [7]. It is widely accepted that both inflammation and impaired energy supply (e.g., mitochondrial dysfunction) play a crucial role in development of NDD [8]. These processes along with the malfunction of specific metabolic pathways may lead to a failure of elimination of proteins from the brain and formation of insoluble plaques [9]. Immune-mediated inflammation characterized by autoreactive lymphocytes in the central nervous system and neurodegeneration with microglial activation and chronic neurodegeneration are also two major pathogenic components in multiple sclerosis [10].

Speculations about an association between achalasia and other autoimmune, demyelinating, or neurodegenerative disorders are not completely new. For example, one recent study found a significant autoimmune co-morbidity among patients with achalasia [11]. However, the association between achalasia and NDD has never been systematically investigated, although there are several publications that point to the co-occurrence of achalasia and an NDD or to finding cellular abnormalities in the digestive tract in patients suffering from an ND [12,13,14,15,16].

We therefore performed a pilot, prospective and questionnaire-based study assessing the association between achalasia and NDD. Our aim was to test the hypothesis that the prevalence of NDD in patients with achalasia (and vice versa) is increased compared to that in general population.

## 2 Patients and methods

### 2.1 Patients

This is a prospective, cross-sectional, questionnaire-based study. Between 2014 and 2019, consecutive patients with confirmed achalasia referred to a tertiary gastroenterology center and patients with confirmed NDD [in our patients, it was multiple sclerosis and clinically isolated syndrome (CIS), Parkinson’s disease, polyneuropathy, spinocerebellar ataxia, motor neuron disease, progressive supranuclear ophthalmoplegia, multisystem atrophy, Allgrove syndrome, epilepsy] at two neurological clinics were offered to participate.

The patients were separately enrolled into two groups-achalasia group (group A) and neurological group (group N). Achalasia had to be diagnosed by clinical evaluation (symptoms), upper GI endoscopy and high-resolution manometry (HRM). Only patients with achalasia according to Chicago classification of motility esophageal disorders were eligible for inclusion. We did not include patients with esophagogastric junction outflow obstruction or an inconclusive HRM finding.

For each patient from group N specific and previously published diagnostic criteria had to be fulfilled. Exclusions criteria for group N were: neurological disorders caused by cancer, stroke, infection, traumatic injury of nervous system and patients in severe condition with life expectancy less than 6 months.


**Ethical approval:** The research related to human use has been complied with all the relevant national regulations, institutional policies and in accordance the tenets of the Helsinki Declaration. The study was approved by the Local Medical Ethics Committees of each participating center.
**Informed consent:** Informed consent has been obtained from all individuals included in this study.

### 2.2 Questionnaires

All included patients were asked to fill in a targeted questionnaire designed specifically for each group to screen for the neurological symptoms in group A and for dysphagia and regurgitation in group N. After inclusion, the patient was automatically excluded from a possible inclusion into the other group.

The questionnaire in the group A (supplementary material) focused principally on the family and personal history of neurodegenerative disorders and on symptoms suggestive of a neurological disease (e.g., history of seizures or loss of consciousness, diplopia, blurred vision, eye pain, visual field defects, color vision deficiency, impaired sensation, postural instability, vertigo, movement disorders, shaking, blood pressure disorders, drop attacks, aphasia or disturbances of smell) (Table 1). If a patient reported any neurological complaint, a thorough neurological examination was carried out including specific diagnostic tests, such as electromyography, somatosensory evoked potentials, motor evoked potentials, electroencephalography, Schirmer test, specific blood tests, brain MRI (magnetic resonance imaging), and lumbar puncture (Table 2).

**Table 1:** The occurrence of neurological symptoms in patients with achalasia (group A)

Symptom	Absolute	Relative (%)
Faintness, loss of consciousness, seizure	20	13.2
Eye pain	19	12.6
Diplopia, blurred vision, visual field defects, color vision deficiency	30	19.9
Impaired sensation	44	29.1
Postural instability, vertigo	24	15.9
Movement disorders, shaking	14	9.3
Blood pressure disorders	70	46.4
Drop attacks, fall for unknown reasons	7	4.6
Aphasia	14	9.3
Disturbance of smell	9	6.0
Impairment of swallowing	133	88.1

**Table 2:** Patients with achalasia (group A) – demographic and clinical data

Patients with achalasia	Group A
Number of patients	150
Gender (M;F)	78;72
Median age (range)	49 (18–83)
Achalasia type I/II/III/not known*	20 (13.3%)/105 (70%)/12 (8%)/13 (8.7%)
Mean IRP (SD); mmHg	27.5 (13.9)
Median Eckardt score (IQR)^a^	7 (5–8)
Positive questionnaire^b^	126 (84.0%)
Presence of neurological symptoms^c^	113 (75.0%)
Neurological examination and specific paraclinical tests^d^	68 (45.0%)
MRI	40 (26.7%)

^a^Eckardt score = validated symptomatic score for achalasia, minimum (no symptoms) 0, maximum 12.

^b^Family and/or personal history was suggestive of possible NDD.

^c^Patients described some neurological symptoms.

^d^Patients with highly suggestive of NDD, performed detailed neurological examination and appropriate diagnostic tests.

*Type of achalasia not reliably established (e.g. HRM was performed after treatment).

IRP = integrated relaxation pressure; SD = standard deviation; IQR = interquartile range; MRI = magnetic resonance imaging.

The questionnaire for patients in the group N (supplementary material) addressed symptoms of achalasia (dysphagia, regurgitation, chest pain) and personal history of esophageal disorders. In patients with clinical suspicion of achalasia, further testing was carried out (upper GI endoscopy, fluoroscopy, HRM) (Table 3).

**Table 3:** Patients with neurodegenerative/demyelinating disorders (group N) - demographic and clinical data

Patients with neurodegenerative/demyelinating disorders	Group N
Number of patients	112
Gender (M;F)	38;74
Median age (range)	50 (18–81)
Dysphagia	32 (28.6%)
HRM (pts. with dysphagia)	16 (72.7%)

HRM = high resolution manometry.

### 2.3 Statistical analysis

We tested for the increased prevalence of NDD in patients with achalasia (group A) and of achalasia in patients with NDD (group N) as compared to the general population using the binomial test. The respective probabilities in the model were given by prevalences in the general population. In the case of achalasia, the general prevalence is known to be 0.8% [17,18]. As the prevalence of NDD in the general population has never been reported, we combined the prevalences of the most common conditions to 1.11% (Table 4) while ignoring polypathologies (co-occurrence of more disorders in a single patient), thus creating an over-estimate. Then, we estimated the total prevalence conservatively to be not greater than 2% (to prevent a false positive result of increased incidence of NDD in patients with achalasia, we had to avoid underestimating the general prevalence of NDD used in the test). We constructed the two-sided 95% confidence interval for the prevalence of NDD in the group A using the Wilson method and calculated the post hoc power using the observed prevalence to be 80%. These measures were not calculated for the prevalence of achalasia in the group N, since the result would be heavily biased (and therefore invalid) due to a small number of patients who underwent HRM examination. The statistical analysis was performed using R version 4.1.3 with the package fastR2 v 1.2.2.

**Table 4:** Prevalence of neurodegenerative/demyelinating disorders in general population [34,35,36,37,38,39,40,41,42,43,44,45]

NDD	Prevalence
Epilepsy	0.008
Parkinson’s disease	0.0015
Multiple sclerosis	0.001
Polyneuropathy (Charcot-Marie-Tooth disease)	0.0003
Huntington’s disease	0.00008
Progressive supranuclear palsy	0.00005
Multiple system atrophy	0.000045
Leber’s optic neuropathy	0.00004
Friedreich ataxia	0.000034
Spinocerebellar ataxia	0.000027
Motor neuron disease	0.00002
Neuroacanthocytosis syndromes	0.00000025
Total estimated	0.02

### 2.4 Main endpoints

The main outcomes of this study were the proportions of patients with achalasia simultaneously suffering from NDD and vice versa. We compared prevalences of NDD/achalasia in the two groups and compared them with those in general population.

## 3 Results

### 3.1 Patients characteristics

From all patients addressed, 50% of respondents consented to participate in the study. A total of 262 patients were enrolled, 150 patients into the group A and 112 into the group N. In the group N, a total of 32 patients reported dysphagia, but only 16 agreed to undergo HRM examination to allow (or rule out) potential diagnosis of achalasia. Tables 2 and 3 show baseline characteristics of patients in both groups.

### 3.2 Prevalence of NDD in patients with achalasia

A diagnosis of NDD was made in 9 of 150 patients [6.0%, 95% confidence interval (CI): 3.1–11.2%] and included: 3 cases of epilepsy, 2 cases of multiple sclerosis, 2 cases of polyneuropathy, 1 case of Leber´s optic neuropathy and 1 case of Parkinson’s disease (Table 5). Thus, we found significantly higher prevalence of NDD in the group A as compared to the estimate of 2% for the general population (*p* = 0.003). Furthermore, 113 (75%) patients reported some neurological symptoms and 21 (14%) patients had a positive family history of a confirmed neurodegenerative or a demyelinating disorder (13 cases of Alzheimer’s disease, 6 cases of multiple sclerosis, 1 case of Leber´s optic neuropathy, 1 case of motor neuron disease and 1 case of Parkinson’s disease).

**Table 5:** Patients with concurrent achalasia and neurodegenerative/demyelinating disorders (group A)

Patient	Year of birth	Confirmation of achalasia (age)	Subtypes of achalasia (Chicago Classification v3.0)	Treatment of achalasia	Neurological disorder	Treatment of neurological disorder
1	1969	47	I	1× pneumatic dilation, 1× POEM	Multiple sclerosis (relapsing-remitting MS)	Interferon beta-1a
2	1973	33	NA	2× pneumatic dilations, 2× injection of botulinum toxin	Multiple sclerosis (relapsing-remitting MS)	Interferon beta-1a
3	1971	42	II	1× POEM	Epilepsy	Valproic acid, carbamazepine
4	1957	54	II	1× POEM	Polyneuropathy	No specific medication
5	1972	38	NA	1× pneumatic dilation, 1× POEM	Epilepsy	Lamotrigine
6	1977	35	II	1× POEM	Leber´s optic neuropathy	No specific medication
7	1986	18	II	1× pneumatic dilation, 1× POEM	Polyneuropathy	No specific medication
8	1932	81	II	1× pneumatic dilation	Parkinson´s disease	Levodopa/carbidopa
9	1958	NA	NA	1× POEM	Epilepsy	Phenytoin, valproic acid

In three patients (No. 2, 5 and 9) we were not able to determine subtype of achalasia due to a previous intervention.

POEM = per-oral endoscopic myotomy; NA = not known or not applicable.

### 3.3 Prevalence of achalasia in patients with NDD

Group N was rather heterogenous regarding the neurological diagnoses (Table 6). The majority of patients (76%) suffered from multiple sclerosis. Among 112 patients, we newly diagnosed 2 patients with achalasia (prevalence 2/112, 1.8%, 1 patient with epilepsy, and 1 patient with polyneuropathy) (Table 7). This prevalence was not significantly increased compared to that in the general population (1.8% vs 0.8% in the general population, *p* = 0.226).

**Table 6:** The NDD diagnoses in the group N

NDD	Absolute (*n* = 112)	Relative (%)
Multiple sclerosis	86	76.8
Polyneuropathy	6	5.4
Epilepsy	6	5.4
CIS^a^	4	3.6
Parkinson’s disease	3	2.7
Spinocerebellar ataxia	2	1.8
Multisystem atrophy	2	1.8
Motor neuron disease	1	0.9
Progressive supranuclear ophthalmoplegia	1	0.9
Allgrove syndrome	1	0.9

^a^CIS refers to a first episode of neurologic symptoms that lasts at least 24 h and is caused by inflammation or demyelination in the central nervous system.

**Table 7:** Patients with NNID and achalasia (group N)

Patient	Year of birth	Confirmation of achalasia (age)	Subtypes of achalasia (Chicago Classification v3.0)	Treatment of achalasia	Neurological disorder	Treatment of neurological disorder
10	1982	26	II	1× POEM	Polyneuropathy	No specific medication
11	1977	4	NA	2× open Heller myotomies	Allgrove syndrome	No specific medication

NA = not known or not applicable.

## 4 Discussion

To our knowledge, this is the first prospective study assessing the prevalence of NDD in patients with achalasia and vice versa. Our study was inspired by our observation of several patients in which achalasia and neurodegenerative disorder occurred simultaneously and of one case of Allgrove syndrome [19].

Our data show an increased prevalence of NDD in patients with achalasia (6%) compared to the estimated prevalence of NDD in general population (2%). On the other hand, we did not observe significantly increased prevalence of achalasia in patients suffering from NDD. Our results suggest a possible overlap between these conditions, which could be a result of some shared pathophysiologic mechanisms.

We also found unexpectedly high proportion of neurologic symptoms and family history of confirmed NDD in patients with achalasia (Table 1); however, in these patients no specific neurological disorder could be diagnosed. The fact that we did not observe an increased prevalence of achalasia in patients suffering from NDD while we did find an increased prevalence of NDD in patients with achalasia might have been caused by the fact that not all patients with dysphagia agreed to undergo esophageal manometry, and, therefore, some patients with real achalasia could have been missed. On the other hand, dysphagia is a frequent symptom in patients with some NDD and it is highly unlikely that a majority of uninvestigated patients had achalasia. In our study, dysphagia was present in 28.6% of patients in group N and this number agrees with published data. For example, Desai and Leland found dysphagia in at least 33% of patients with impaired multiple sclerosis [15].

Our analysis was based on the estimated prevalence of achalasia and NDD in the general population. As there is an agreement with regard to the prevalence of achalasia in the general population [17,18], the situation in terms of NDD is more complicated. Classification of neurodegenerative/demyelinating disorders differs among different centers and their prevalence is a matter of discussion, especially in the case of rare diseases. In order to estimate the overall prevalence of NDD, we first summed the prevalence of disorders listed in Table 4 to 1.11%, and then we created an upper estimate reflecting all the non-listed rare disorders. Thus, our 2% estimation (used for testing) includes a safe margin, and it is extremely unlikely that the overall prevalance of NDD would be higher.

Achalasia and NDD share some common pathophysiological mechanisms, even if the exact pathogenesis is unknown. Both achalasia and some NDD may be caused by autoimmune mechanisms. Speculations about the autoimmune pathogenetic pathway in achalasia is based on the consistent finding of inflammatory changes in the histological specimen from the esophageal tissue including T cells, autoantibodies, and complement activation in the ganglia of the myenteric plexus [20]. Also, in some cases of paraneoplastic pseudoachalasia, circulating non-specific and specific (anti-Hu) antibodies were observed [21,22,23]. Moreover, patients with achalasia are 3.8× more likely to suffer from other autoimmune diseases compared to patients with gastroesophageal reflux disease [11]. Involvement of autoimmune processes is supported by a recent finding of an association of achalasia with a genetic variant in HLA-DQ region in chromosome 6 [4]. The alternative hypothesis how achalasia develops is so-called neurodegenerative theory, which is supported by the presence of Lewy bodies in the cells of myenteric plexus and degeneration of neurons in dorsal motor nucleus and vagal nerves in patients with achalasia [24,25]. In addition, autoimmune process and/or neurodegeneration might be triggered by viral infection. Of course, both autoimmunity and neurodegeneration are the main pillars of the development of NDD, and in some disorders, the influence of infection (most often viral) cannot be ruled out. Based on these assumptions, association between achalasia and NDD may be plausible. Beside our results, this association is further supported by the following findings: (1) co-occurrence of achalasia and central nervous system degeneration [12,14], multiple sclerosis [15], or peripheral nervous system inflammation (e.g. Guillain-Barré syndrome) [11,16,26]; (2) myenteric plexus degeneration and accumulation of insoluble proteins found throughout the digestive tract in patients with neurodegenerative or neuroinflammatory disorders of the central nervous system (e.g., Parkinson´s disease) [27,28,29,30]; (3) immunohistochemistry studies suggesting common pathophysiological pathways (oxidative stress and ganglionic degeneration) in both central and enteric neurons in patients with NDD [27,28]; and (4) geospatial gradient from South to North and variants in the HLA region with increasing risk of both multiple sclerosis and achalasia [31,32,33].

Our study has several limitations. First, the overall prevalence of all included NDDs in the general population is not known and we had to estimate their total prevalence conservatively to compare it to the prevalence among patients with achalasia, thus we potentially reduced the power of the test. Second, relatively small groups of patients with miscellaneous NDDs were recruited and only sixteen patients with NDD and dysphagia agreed to undergo HRM to prove a potential esophageal motility disorder. Thus, because of a high proportion of patients who declined HRM only a lower bound for the prevalence of achalasia among NDD patients could be assessed. Assuming another two cases of achalasia among the other 16 HRM un-examined patients with dysphagia, we would obtain a significantly increased prevalence of achalasia (4/112 = 3.6% versus 0.8%, *p* = 0.013). Unfortunately, having made the consent to HRM an inclusion criterium for the whole study could, on the other hand, have induced a selection bias.

## 5 Conclusion

Our study suggests higher prevalence of NDDs in patients with achalasia (6%) than in general population (2%). Therefore, the proactive search and treatment of an NDD in patients with esophageal achalasia might be recommended. Nevertheless, our preliminary results warrant confirmation in a large population-based study. Further investigation of possible shared etiopathogenetic traits may help to better understand the complex etiology of achalasia, which might lead to changing its treatment strategy from the symptomatic approach to the causal therapy.
